# A prognostic model and pre-discharge predictors of post-COVID-19 syndrome after hospitalization for SARS-CoV-2 infection

**DOI:** 10.3389/fpubh.2023.1276211

**Published:** 2023-11-29

**Authors:** Oleksii Honchar, Tetiana Ashcheulova, Tetyana Chumachenko, Dmytro Chumachenko, Alla Bobeiko, Viktor Blazhko, Eduard Khodosh, Nataliia Matiash, Tetiana Ambrosova, Nina Herasymchuk, Oksana Kochubiei, Viktoriia Smyrnova

**Affiliations:** ^1^Department of Propedeutics of Internal Medicine No.1, Fundamentals of Bioethics and Biosafety, Kharkiv National Medical University, Kharkiv, Ukraine; ^2^Department of Epidemiology, Kharkiv National Medical University, Kharkiv, Ukraine; ^3^Department of Mathematical Modelling and Artificial Intelligence, National Aerospace University "Kharkiv Aviation Institute", Kharkiv, Ukraine; ^4^Department of Pulmonology, MNE “Clinical City Hospital No.13” of Kharkiv City Council, Kharkiv, Ukraine

**Keywords:** COVID-19, hospitalization, convalescence, post-acute COVID-19 syndrome, walk test, echocardiography, clinical decision rules, machine learning

## Abstract

**Background:**

Post-COVID-19 syndrome (PCS) has been increasingly recognized as an emerging problem: 50% of patients report ongoing symptoms 1 year after acute infection, with most typical manifestations (fatigue, dyspnea, psychiatric and neurological symptoms) having potentially debilitating effect. Early identification of high-risk candidates for PCS development would facilitate the optimal use of resources directed to rehabilitation of COVID-19 convalescents.

**Objective:**

To study the in-hospital clinical characteristics of COVID-19 survivors presenting with self-reported PCS at 3 months and to identify the early predictors of its development.

**Methods:**

221 hospitalized COVID-19 patients underwent symptoms assessment, 6-min walk test, and echocardiography pre-discharge and at 1 month; presence of PCS was assessed 3 months after discharge. Unsupervised machine learning was used to build a SANN-based binary classification model of PCS development.

**Results:**

PCS at 3 months has been detected in 75% patients. Higher symptoms level in the PCS group was not associated with worse physical functional recovery or significant echocardiographic changes. Despite identification of a set of pre-discharge predictors, inclusion of parameters obtained at 1 month proved necessary to obtain a high accuracy model of PCS development, with inputs list including age, sex, in-hospital levels of CRP, eGFR and need for oxygen supplementation, and level of post-exertional symptoms at 1 month after discharge (fatigue and dyspnea in 6MWT and MRC Dyspnea score).

**Conclusion:**

Hospitalized COVID-19 survivors at 3 months were characterized by 75% prevalence of PCS, the development of which could be predicted with an 89% accuracy using the derived neural network-based classification model.

## 1 Background

Since 2019–2020, the novel SARS-CoV-2 infection has emerged as a global health challenge. During the first year of COVID-19 pandemic, the cumulative effect of overwhelming of healthcare systems, lack of effective etiological treatment, and more severe course of disease that was typical to early SARS-CoV-2 variants had led to high morbidity and significant excess mortality (1), and the cascade of prolonged lockdowns that were introduced in an attempt to slow down the epidemic process imposed an additional strain on economies worldwide (2). Subsequent success of the global vaccination campaign has dramatically reduced the prevalence of severe and life-threatening cases whilst imposing less significant initial effect on the morbidity (3). Moreover, gradual lifting of lockdowns contributed to multifocal re-acceleration of the COVID-19 spread, with the daily new cases count in January 2022 exceeding the parameters during previous peaks more than three-fold (4). As a result, the current phase of lower morbidity that we observe since the beginning of 2023 is being continuously accompanied by an increasing number of patients suffering from long lasting sequelae of SARS-CoV-2 infection.

Unlike in most other respiratory viral infections, persistence of symptoms after the end of the acute phase is highly prevalent in COVID-19 convalescents (5, 6) and is shown to be associated with a decreased quality of life (7). Potential pathogenetic mechanisms are heterogenous and include consequences of direct organs injury during the acute phase, persistent SARS-CoV-2 replication, dysautonomia, altered immune reactivity, coagulopathy, endotheliopathy, and gastrointestinal microbiome disturbances (8, 9). “Long COVID,” the most popular term reserved for this scenario, accounts for all manifestations that persist beyond 4 weeks after disease onset and, therefore, includes the early post-acute period of 4 to 12 weeks (also referred to as “ongoing symptomatic COVID-19”), when one expects to observe good natural dynamics of residual symptoms resolving in a significant proportion of patients (10). At the same time, development of the “post-COVID-19 syndrome,” being defined as persistence of symptoms beyond 12 weeks, presents a more significant socio-economic problem, given the debilitating effect of some of the most prevalent symptoms, such as fatigue, dyspnea, apathy, and cognitive dysfunction (6, 10, 11).

The prevalence of post-COVID-19 syndrome (PCS) ranges from 5 to 37% in the general population of convalescents but may reach as high as 76 to 81% in those who required hospitalization (6, 12). To date, cardiopulmonary rehabilitation remains the mainstay of management of these patients, with locally available resources being at times insufficient to deal with the total flow of new convalescents (13–15). In this setting, creation of tools to assess the risk of subsequent development of PCS basing on pre-discharge data might be used to optimize the selection of candidates for supervised rehabilitation programs following hospitalization.

### 1.1 Objective

To study the in-hospital clinical characteristics of COVID-19 survivors presenting with self-reported post-COVID-19 syndrome at 3 months and to identify the early predictors of its development.

## 2 Materials and methods

### 2.1 Study design and population

By design, this is a cross-sectional prospective observational single-center study that was performed at Kharkiv City Hospital No.13 that at the time served as a specialized tertiary COVID-19 care center. Between January and November 2021, patients that were hospitalized with the diagnosis of pneumonia were evaluated for eligibility criteria that included the age of ≥18 years and positive polymerase chain reaction test for SARS-CoV-2. In total, 265 consecutive eligible patients were identified and invited to participate in the study; 44 of those have declined participation, and 221 were enrolled. The final cohort included 166 patients who have completed the repeated visit at 3 months post-discharge – see Supplementary Figure S1 for the study flowchart.

The study was conducted in compliance with the standards of Helsinki Declaration and was approved by the ethical committee of Kharkiv National Medical University (No. 3/2021). All participants provided written informed consent.

### 2.2 Clinical data collection

The first visit was performed pre-discharge, in clinically stable patients who were meeting clinical criteria of epidemic safety [resting capillary blood oxygen saturation (SpO2) >93% on room air, absence of acute respiratory symptoms and normal body temperature for ≥3 days starting from the 10th day after the onset of disease (16)] and included the analysis of medical records to collect data on patients’ age, gender, laboratory and instrumental findings, and treatment used; an interview to obtain data on symptoms and medical history; and anthropometry.

Transthoracic echocardiography (TTE) was performed by an expert physician who was blinded to patients’ data prior to procedure, using Radmir ULTIMA ultrasound system (Radmir Co., Ukraine). The measurements were performed in strict accordance with the respective guidelines by the American Society of Echocardiography (ASE) and European Association of Cardiovascular Imaging (EACVI) (17–19) and included cardiac chambers morphometry (left atrial and left ventricular [LV] volumes, right atrial area, right ventricular size, relative walls thickness and myocardial mass index of the LV), assessment of the indices of systolic function (LV ejection fraction using Simpson biplane method, mitral and tricuspid annular planes systolic excursion, global longitudinal strain [GLS] of ventricles using the linear method (20–22), and mitral annular s’ velocity in the tissue Doppler mode) and LV diastolic function (mitral e’ velocity and E/e’ ratio).

6-min walk test (6MWT) was performed by a physician using the standard methodology as recommended by the American Thoracic Society guidelines (23), in a self-paced mode, with no use of practice tests, warm-up or non-standardized encouragement. A 20-m hallway was used, determining the selection of models to calculate the individual predicted distance (24). Pulse rate and SpO2 were registered at start and every 30 s thereafter using a Bluetooth-connected pulse oximeter; levels of fatigue and dyspnea were assessed at the baseline and at finish using modified Borg scale. Along with 6-min walk distance (6MWD), reached percent of predicted distance (6MWD%), baseline and minimal capillary oxygen saturation (SpO2base and SpO2min), baseline and maximal heart rate (HRbase and HRmax) were analyzed. Peak oxygen desaturation was calculated as SpO2drop = SpO2min - SpO2base, and reached percent of the predicted maximal heart rate as HRmax% = 100% × HRmax / (208–0.8 × Age) (25).

A follow-up visit at 1 month after discharge followed the same protocol, and the final visit at 3 months was performed distantly (by means of telephone, email, and text messengers) and included re-assessment of symptoms and detection of the PCS which was defined as a self-reported perceived worsening of health status compared to the pre-COVID-19 state or persistence of new symptoms during the last month before the visit.

### 2.3 Statistical analysis

The data analysis was performed using StatSoft STATISTICA Version 12 software package. Shapiro–Wilk test was used to assess the distribution of data. Continuous variables are reported as mean ± standard deviation (SD) in case of normal distribution and as median [interquartile range] in case of skewed distribution. Categorical variables are reported as counts (percentages). Independent samples t-test was used to compare normally distributed continuous variables, and paired samples t-test was used for longitudinal comparisons. For skewed variables, the comparisons were made using Mann–Whitney U-test or Wilcoxon signed-rank test. Binary and categorical variables were compared using Chi-Square test. The differences were considered significant if *p* < 0.05. Marginal effects of potential PCS predictors in logistic regression analysis were used as a measure to select inputs for unsupervised machine learning based training of simple artificial neural networks (SANN). Random sampling was used to select training, test and validation subsets of the study cohort in the 70:15:15 proportion. For each set of input variables, 500 binary classification models were trained. This number represented an empirical balance between computational resource allocation and model reproducibility – fluctuating prediction accuracy was observed with fewer models, whereas the consistency in results was achieved at n = 500; further increasing the model count did not yield significant improvements but incurred greater computational time. Automated neural architecture search strategy and Broyden-Fletcher-Goldfarb-Shanno optimization algorithm were used; missing data was imputed by mean values. Predictive performance of the obtained models was assessed as percentage of correctly classified cases from the test and validation subsets. For the final predictive model, 10-fold cross-validation was used to ensure its reproducibility, and ROC analysis performed. A post-hoc approach incorporating assessment of the model accuracy and the dataset effect size using Cohen’s d statistic was used to evaluate the sample size adequacy (26).

## 3 Results

### 3.1 Baseline characteristics

The final study cohort included 76 male and 90 female patients at the mean age of 53.7 ± 13.3 years. Visit 1 was performed at the median of 22 days, visit 2 at 53 days, and visit 3 at 116 days after the symptoms onset. Among the observed cohort, 124 (75%) were reporting ongoing new symptoms and/or self-estimated worsening of health status that was classified as post-COVID-19 syndrome. The comparative clinical characteristic of study participants with and without ongoing symptoms at 3 months is presented in Table 1. The patients with PCS were older, more frequently female, had higher BMI and comorbidities burden. In their in-hospital laboratory profile, patients with ongoing symptoms at 3 months had higher values of C-reactive protein, erythrocyte sedimentation rate, and higher proportion of patients with very high interleukin-6 values, which attested to higher inflammatory activity. The observed lower values in estimated glomerular filtration rate were explained by age and gender differences. There were no differences in received treatment, but PCS patients more frequently required oxygen support during hospitalization.

**Table 1:** Demographics and pre-discharge clinical characteristics of the study participants with and without post-COVID-19 syndrome.

Parameters	No post-COVID-19 syndrome	Post-COVID-19 syndrome	Difference (95% CI)	2-sided *p*
Subjects	42	124		
Female sex	16 (38)	74 (60)		0,015
Age, years	48,7 ± 17,0	55,4 ± 12,1	6,7 (1,9; 11,5)	0,006
BMI, kg/m^2^	27,3 ± 4,9	29,6 ± 5,2	2,3 (0,5; 4,1)	0,012
Comorbidities				
Hypertension Obesity Diabetes mellitus, type 2 History of peptic ulcer History of cancer History of stroke / TIA Chronic kidney disease Bronchial asthma COPD Angina pectoris Chronic liver disease Charlson comorbidity index	15 (36) 10 (24) 2 (5) 0 (0) 0 (0) 0 (0) 0 (0) 0 (0) 1 (2) 0 (0) 0 (0) 0,19 ± 0,40	50 (40) 51 (41) 15 (12) 12 (10) 7 (6) 6 (5) 4 (3) 4 (3) 2 (2) 3 (2) 2 (2) 0,57 ± 0,82		0,5970,044 0,175 0,036 0,116 0,147 0,239 0,239 0,747 0,309 0,408 0,005
Active smoking status	7 (17)	16 (13)		0,542
Pulmonary involvement by CT, %	37,8 ± 25,5	30,6 ± 18,7	−7,2 (−17,0; 2,6)	0,147
Minimal in-hospital SpO2, %	90,0 ± 6,4	87,6 ± 7,9	−2,4 (−5,1; 0,2)	0,072
Oxygen supplementation				
Via nasal cannula Noninvasive/invasive ventilation	16 (38) 4 (10)	77 (62) 5 (4)		0,0070,174
Laboratory parameters				
Peak IL-6, pg./mL Peak CRP, mg/L Peak ESR, mm/h Peak procalcitonin, ng/mL Peak D-dimer, ng/mL Peak creatinine, μmol/L Lowest eGFR, ml/min/1,73m^2^ Hemoglobin, g/dL	8,6 [3,3; 11,7]11 [6; 27]26,8 ± 10,6 0,06 [0,05; 0,11] 323 [199; 432] 99,0 ± 21,0 76,9 ± 25,1 14,1 ± 1,7	11,7 [3,0; 47,0]25 [12; 74]32,2 ± 13,0 0,06 [0,04; 0,12] 279 [156; 508] 104,6 ± 23,0 60,7 ± 13,7 13,8 ± 1,5	13,4 (0,2; 26,7)27,7 (0,0; 55,3) 5,4 (0,2; 10,5) 0,3 (−0,7; 1,2) −18 (−253; 217) 5,5 (−4,7; 15,8) −16,2 (−24,2; 8,2) −3,1 (−9,4; 3,3)	0,4580,007 0,044 0,750 0,524 0,285 < 0,001 0,342
Treatment				
Dexamethasone Methylprednisolone Remdesivir	40 (95)28 (67) 21 (50)	107 (86)83 (67) 53 (43)		0,1150,975 0,413

BMI, body mass index; TIA, transient ischemic attack; COPD, chronic obstructive pulmonary disease; CT, computed tomography; SpO2, peripheral capillary oxygen saturation; IL-6, interleukin 6; CRP, C-reactive protein; ESR, erythrocyte sedimentation rate; eGFR, estimated glomerular filtration rate by CKD-EPI equation.

### 3.2 Physical performance assessment

Analysis of the 6MWT parameters (see Table 2) has revealed that the apparent decrease of 6-min walk distance (6MWD) that was observed pre-discharge in PCS patients was explained by age and sex differences, evidenced by close values of the reached percent of individually predicted distance (6MWD%). Moreover, the PCS group has paradoxically demonstrated larger between-visits increment of both parameters, resulting in higher 6MWD% values at 1 month. At the same time, the level of subjective symptoms in these patients was significantly higher at visit 2, being explained by much worse improvement from the pre-discharge baseline compared to non-PCS participants. This difference was not explained by the observed values of capillary oxygen saturation throughout the test (there was no difference between groups), nor could it be attributed to worse dysautonomia – despite initially lower heart rate (HR) increment during the test at visit 1, the PCS patients have demonstrated better dynamics of utilization of HR reserve, which resulted in higher reached percent of the individual HR maximum at 1 month. MRC dyspnea scale score at both visits was also higher in PCS participants with the mean values of 2.5 ± 1.1 vs. 2.0 ± 1.0 pre-discharge (*p* = 0.005) and 1.8 ± 0.8 vs. 1.3 ± 0.6 at visit 2 (*p* = 0.002).

**Table 2:** One-month post-discharge dynamics of 6-min walk test parameters in patients with and without post-COVID-19 syndrome.

Parameters	No post-COVID-19 syndrome	Post-COVID-19 syndrome	Difference (95% CI)	2-sided *p*
Distance: 6MWD at Visit 1, m 6MWD% at Visit 1, % 6MWD at Visit 2, m 6MWD% at Visit 2, % Delta 6MWD between visits, m Delta 6MWD% between visits, %	444 ± 57 62,5 ± 9,8 490 ± 64 68,2 ± 11,6 49 ± 33 7,3 ± 5,0	380 ± 64 62,5 ± 9,6 454 ± 63 75,1 ± 9,5 77 ± 44 13,4 ± 7,1	−64 (−90; −38) 0,0 (−4,1; 4,0) −36 (−65; −8) 6,9 (2,4; 11,3) 28 (9; 46) 6,1 (3,0; 9,1)	< 0,001 0,986 0,012 0,003 0,004 < 0,001
Heart rate: HRbase at Visit 1, bpm HRmax at Visit 1, bpm HRmax% at Visit 1, % HRrise at Visit 1, bpm HRbase at Visit 2, min^−1^ HRmax at Visit 2, min^−1^ HRmax% at Visit 2, % HRrise at Visit 2, bpm	81,1 ± 11,0 109,0 ± 9,2 61,2 ± 6,0 26,7 ± 9,7 78,6 ± 15,7 108,2 ± 11,3 60,7 ± 5,8 29,1 ± 9,9	83,3 ± 13,2 105,0 ± 15,6 60,4 ± 12,5 21,5 ± 11,5 82,2 ± 13,5 110,5 ± 14,6 65,3 ± 8,3 27,7 ± 12,1	2,2 (−2,8; 7,2) −4,0 (−10,4; 2,4) −0,8 (−5,7; 4,0) −5,2 (−10,1; −0,3) 3,6 (−2,5; 9,8) 2,4 (−3,8; 8,6) 4,6 (1,1; 8,1) −1,4 (−6,6; 3,9)	0,388 0,216 0,734 0,039 0,239 0,451 0,010 0,608
Oxygen saturation: SpO2base at Visit 1, % SpO2min at Visit 1, % SpO2drop at Visit 1, % SpO2base at Visit 2, % SpO2min at Visit 2, % SpO2drop at Visit 2, %	95,2 ± 5,6 94,7 ± 3,9 2,5 ± 2,5 98,2 ± 0,6 95,7 ± 2,8 2,5 ± 2,8	95,1 ± 5,2 93,8 ± 4,2 3,1 ± 2,7 98,0 ± 0,8 95,8 ± 2,1 2,2 ± 1,9	−0,1 (−2,0; 1,8) −0,9 (−2,6; 0,8) 0,6 (−0,5; 1,7) −0,2 (−0,6; 0,1) −0,1 (−0,9; 1,1) −0,3 (−1,3; 0,6)	0,887 0,293 0,317 0,155 0,837 0,520
Symptoms*: Dyspnea at start, Visit 1, pts. Fatigue at start, Visit 1, pts. Dyspnea at finish, Visit 1, pts. Fatigue at finish, Visit 1, pts. Dyspnea at start, Visit 2, pts. Fatigue at start, Visit 2, pts. Dyspnea at finish, Visit 2, pts. Fatigue at finish, Visit 2, pts	1,1 ± 1,3 2,1 ± 1,6 2,6 ± 1,8 3,1 ± 2,4 0,4 ± 0,8 0,8 ± 1,2 1,3 ± 1,0 0,9 ± 1,0	1,4 ± 1,6 2,5 ± 2,3 3,8 ± 1,9 3,5 ± 1,8 0,9 ± 1,1 1,5 ± 1,3 3,2 ± 1,7 2,9 ± 1,9	0,3 (−0,3; 1,0) 0,4 (−0,5; 1,3) 1,2 (0,1; 2,3) 0,4 (−0,7; 1,4) 0,5 (0,1; 1,0) 0,7 (0,1; 1,3) 2,0 (1,1; 2,8) 2,0 (1,1; 2,9)	0,346 0,379 0,027 0,482 0,022 0,014 < 0,001 < 0,001

*Assessed using modified Borg scale; CI, confidence interval; 6MWD, 6-min walk distance; 6MWD%, reached % of predicted 6-min walk distance; HRbase, baseline heart rate; HRmax, maximal reached heart rate; HRmax%, reached percent of the individual maximum heart rate; SpO2base, baseline oxygen saturation; SpO2min, minimal oxygen saturation; SpO2drop, peak oxygen desaturation.

### 3.3 Echocardiographic assessment

Retrospective assessment of echocardiographic features of observed patients has only revealed minor differences between PCS and non-PCS study participants (see Table 3). Both groups were showing a strong trend to concentric LV remodeling [refer to (27) for the detailed comparison to matched control], and patients who were subsequently reporting long-lasting symptoms had somewhat smaller ventricular cavities. Despite this fact, systolic atrioventricular annuli excursion was comparable to PCS-free patients, translating into somewhat higher longitudinal strain values (reaching statistical significance in case of RV). The borderline changes in diastolic LV parameters became more apparent at visit 2, with significantly lower e’ velocity (9.4 ± 2.6 vs. 10.7 ± 3.1 cm/s, *p* = 0,025) and higher E/e’ ratio (7.6 ± 2.4 vs. 6.4 ± 1,6, p = 0,020) being observed in the PCS group; the only other difference that persisted at 1 month was the slightly higher RV free wall strain values in the PCS cohort.

**Table 3:** Pre-discharge echocardiographic characteristic of hospitalized COVID-19 patients.

Parameters	No post-COVID-19 syndrome	Post-COVID-19 syndrome	Difference (95% CI)	2-sided *p*
Left chambers morphometry				
LA volume index, ml/m^2^	28,7 ± 7,7	28,7 ± 6,3	0,1 (−2,3; 2,4)	0,961
LV end-diastolic volume index, ml/m^2^	51,2 ± 8,2	47,5 ± 9,2	−3,8 (−6,9; −0,6)	0,020
LV end-systolic volume index, ml/m^2^	17,8 ± 5,0	16,4 ± 4,7	−1,4 (−3,1; 0,2)	0,093
LV relative wall thickness	0,44 ± 0,07	0,46 ± 0,08	0,01 (−0,01; 0,04)	0,298
LV mass index (height^2,7^), g/m^2,7^	38,5 ± 10,7	37,8 ± 8,6	−0,7 (−3,9; 2,6)	0,674
Left ventricular function				
LV ejection fraction, %	65,3 ± 7,5	65,5 ± 6,6	0,2 (−2,3; 2,6)	0,900
Lateral MAPSE, mm	15,2 ± 2,8	15,2 ± 2,2	0,0 (−0,1; 0,1)	0,961
LV global longitudinal strain, %	16,9 ± 2,3	17,6 ± 2,4	0,7 (−0,2; 1,6)	0,137
LV s’, cm/s	9,8 ± 1,6	9,7 ± 1,8	−0,1 (−0,7; 0,5)	0,787
LV e’, cm/s	9,7 ± 2,2	9,0 ± 2,2	−0,7 (−1,5; 0,1)	0,070*
LV E/e’ ratio	7,4 ± 2,0	7,5 ± 1,7	0,1 (−0,5; 0,7)	0,763
Right chambers evaluation				
RA area index, cm^2^/m^2^	8,1 ± 1,1	8,2 ± 2,4	0,0 (−0,8; 0,9)	0,908
RV size (proximal outflow tract), mm	31,2 ± 3,7	32,0 ± 3,2	0,8 (−0,5; 2,1)	0,241
RV longitudinal size, mm	70,7 ± 5,9	67,6 ± 7,4	−3,1 (−5,8; −0,5)	0,021
TAPSE, mm	24,5 ± 4,9	25,0 ± 4,1	0,4 (−1,2; 2,1)	0,604
RV free wall longitudinal strain, %	33,5 ± 5,0	37,2 ± 7,1	3,7 (0,9; 6,4)	0,009

*1-sided *p* = 0,034; CI, confidence interval; LA left atrium; LV, left ventricle; MAPSE, mitral annular plane systolic excursion; RA, right atrium; RV, right ventricle; TAPSE, tricuspid annular plane systolic excursion.

### 3.4 Prediction of post-COVID-19 syndrome development

To create a tool that would predict the PCS development, unsupervised machine learning was used to train SANN-based binary classification models. In order to avoid overfitting effect in the setting of a relatively small sample size, we applied a two-step approach, where the list of potential inputs was first narrowed down using the assessment of their marginal effects in the logistic regression analysis. In the order of decreasing prognostic value, significant pre-discharge predictors included pre-discharge 6MWD, eGFR, heart rate increment during 6MWT, SBP, RV free wall strain, ESR, LV end-diastolic index, BMI, in-hospital oxygen supplementation, Charlson’s comorbidity index, height, sex, age, and obesity (see Supplementary tables 1–2 and Supplementary figure 2 for exact Somers’ D values and the resulting regression model parameters). As a next step, identified parameters were used as inputs for SANN-based classification model.

**Figure 1:**
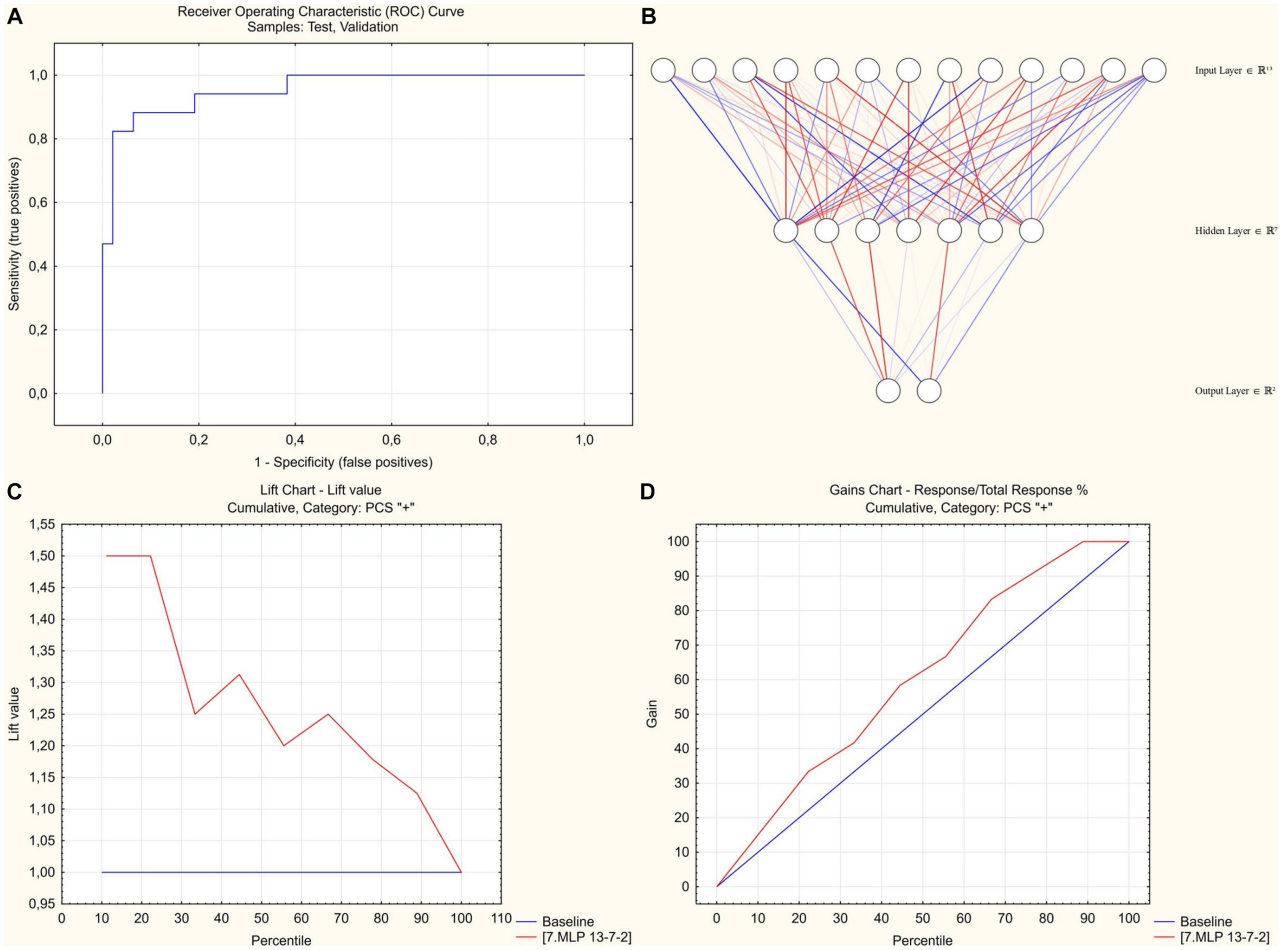
SANN-based classification model to predict post-COVID-19 syndrome at 3 months post-discharge. **(A)** Receiver operator characteristic analysis. Area under curve = 0,955. **(B)** 13–7-2 SANN architecture. **(C)** Lift chart for predicting PCS-positive cases. **(D)** Gains chart for predicting PCS-positive cases.

The use of the complete set of significant pre-discharge predictors, however, did not result in generation of high-performance models: the best predictive accuracy in the test/validation subset was 83%, which (given the 75% prevalence of PCS) meant that only 1/3 of otherwise false-positive cases could be correctly reclassified compared to the blunt assumption that all participants will develop the PCS. Addition of the parameters obtained during the first follow-up visit that was performed 1 month after discharge (see Supplementary Table 3 for marginal effects in logistic regression analysis) with subsequent stepwise deletion of excessive variables based on the results of global sensitivity analysis of the current version of the model have resulted in creation of the model that was characterized by a 93% predictive performance in the training and 89% in the randomly selected test/validation subsets of the study group (91% for sensitivity, 82% for specificity). The model utilized 13–7-2 SANN architecture and used age, sex, in-hospital levels of CRP, eGFR and need for oxygen supplementation, and level of post-exertional symptoms at 1 month after discharge (fatigue and dyspnea in 6MWT and MRC Dyspnea score) as inputs (Figure 1; see Supplementary Table S4 for network weights and connections; source file available at https://doi.org/10.5281/zenodo.8395451). 10-fold cross-validation that was performed by re-training the neural networks using alternative sampling has allowed to consistently obtain non-inferior accuracy in the test/validation subsets.

## 4 Discussion

Despite the end of the COVID-19-related public health emergency that was declared by the World Health organization on May 5, 2023, it remains an ongoing global health issue (28). Moreover, asynchronous trends of its spread and non-uniform levels of vaccination worldwide contribute to the possibility of persistence of epidemic process in separate areas, being associated with a potential for selection of new SARS-CoV-2 variants. Another issue of growing concern is the frequent transition of symptomatic COVID-19 to the chronic phase. Moreover, the recent large meta-analysis shows that the unusually high incidence of post-acute sequelae as assessed at 1 to 3 month after onset of disease (6, 12) is translating into a long-lasting trail of impaired health status: 50% of COVID-19 survivors continue to report at least one new symptom 1 year after acute infection, with the most typical manifestations (such as fatigue, dyspnea, psychiatric symptoms, cognitive deficit, memory impairment) having a potential to impose a long-term debilitating effect (29). Thus, despite having hopefully stabilized the acute COVID-19 problem, we are currently facing a new protracted global health challenge presented by the post-COVID-19 syndrome, and the timely identification of patients at risk of its development might be instrumental for subsequent targeted attempts to improve their expected poor trajectory.

The current study was focused on the assessment of pre- and early post-discharge characteristics of hospitalized COVID-19 survivors who presented with ongoing symptoms or a self-reported persistent decline in the general health status at 3 months after discharge. Compared to the patients who have completely recovered by this term, subjects with PCS were characterized by older age, higher BMI, were more frequently female, had higher burden of comorbidities and more intensive inflammatory response during the acute phase, as evidenced by higher values of CRP, ESR, and larger share of patients with very high IL-6 levels. Despite non-inferior physical functional status as assessed by the 6MWT, PCS patients were reporting higher levels of fatigue and dyspnea, which became more pronounced at 1 month post-discharge due to worse dynamics of improvement compared to symptom-free individuals. Considering an insignificant difference in the minimal SpO2 levels between groups, it was most likely the higher level of subjective symptoms that was driving more frequent in-hospital oxygen supplementation in the PCS cohort. Comprehensive TTE has revealed similar changes in both groups that persisted at 1 month and foremost included a high incidence of concentric LV remodeling and grade I diastolic dysfunction; PCS patients were characterized by smaller ventricular cavities pre-discharge and worse diastolic filling at 1 month but higher RV longitudinal strain throughout the period of follow-up.

Compared to the set of predictors of poor physical functional recovery at 1 month post-discharge in the same cohort of patients (30), age, ESR, eGFR, need for in-hospital oxygen supplementation, and pre-discharge 6MWD have retained their prognostic value for predicting the outcome at 3 months, whereas the extent of radiological pulmonary involvement, pre-discharge SpO2, and history of hypertension became insignificant. At the same time, we revealed a subset of additional predictors of PCS that were either irrelevant (sex, height, BMI, SBP) or had not been analyzed at 1 month (RV free wall strain, LV end-diastolic index, and Charlson’s comorbidity index).

Despite the large number of studies that assessed the epidemiology of PCS, the data on its predictors differ according to different sources, and the strength of observed associations is frequently weak (29). Among the variety of potential predictors, the few that have been identified regularly in different studies include older age (31, 32), history of smoking and/or lung disease (31, 33, 34), disease severity (31, 33, 35), longer hospital stay (35, 36), higher levels of CRP (32, 37), severity of symptoms (36), grade of pulmonary CT changes (35, 38), and requirement of in-hospital and post-discharge oxygen support (34, 35, 39), which puts the results of our study in line with the available body of knowledge.

Despite the poor pre-discharge results of 6MWT and evidence of incomplete early physical functional recovery, we did not find these parameters to be independently predictive of preserved self-reported COVID-19 sequelae at 3 months when accounted for sex, age, and anthropometrical parameters, thus confirming the conclusion of Ladlow et al. (40) about the lack of association between the objective functional limitations and the level of residual symptoms in the setting of PCS. We also have not found significant differences in cardiac structure and function both pre-discharge and after a one-month follow-up: patients with and without the PCS seemed to express similar changes that were in line with those reported before (41, 42).

The difficulties in predicting the development of post-COVID-19 syndrome might be related to the heterogenous underlying mechanisms, differences in vaccination status at the population level, and the constantly changing landscape of temporarily and locally prevailing SARS-CoV-2 variants. The literature search has allowed us to identify several finished studies proposing tools for predicting the outcomes of the acute phase of disease (43), effects of post COVID-19 rehabilitation (44), development of post-acute cardiovascular complications (45), and dynamics of the radiological recovery (46). Additionally, we have identified a published protocol of the study directed at solving the problem of predicting the development of the long COVID syndrome (47). To our knowledge, the current study is the first to date presenting a classification model that allows to predict the development of self-reported post-COVID-19 syndrome based on the acute phase parameters in the cohort of hospitalized patients that were shown to be at higher risk of PCS.

Logistic regression analysis is a traditional first line tool to solve the binary classification tasks; at the same time, it is vulnerable to overfitting when using smaller datasets (n < 500) (48). Machine learning approach in our study has allowed to overcome this obstacle; minimization of the input variables number led the training subset to meet the usual sample size requirements for ML projects with a cases-to-predictors ratio of 15.4:1 (49). Post-hoc analysis involving the input dataset effect size (Cohen’s d statistic = 0,684) and the model’s predictive accuracy of 89% additionally confirmed the appropriateness of the sample size (26).

### 4.1 Limitations of the study

The limitations of our study include possible center-related effects, potential selection bias related to inclusion of less severe cases as a result of inability of persistently O2-dependent patients to participate, negligible level of vaccination in the study population and different prevailing SARS-CoV-2 variants at the time of enrollment compared to today. Hence, the results of the study should be cautiously generalized to the current post-COVID-19 care practice. Given the ever-changing landscape of the acute SARS-CoV-2 infection setting, any types of newly developed predictive tools would require an external validation on the current local cohorts of COVID-19 convalescents as a part of their proper implementation. The proposed model should, therefore, be perceived both as a potential ready-to-use predictive tool and as a proof of concept for the development of similar models based on the more recent data from local populations.

## 5 Conclusion

Hospitalized patients with SARS-CoV-2 infection were characterized by a 75% prevalence of post-COVID-19 syndrome at 3 months after discharge, with PCS subjects being older, more frequently female, having higher BMI, more intensive acute inflammatory response, and lower eGFR. Higher level of symptoms in the PCS group was not associated with worse physical functional recovery or significant changes on TTE compared to symptoms-free participants. Despite identification of a set of pre-discharge predictors, inclusion of parameters obtained at 1 month proved necessary to obtain a high accuracy ML-based classification model of PCS development; the final list of inputs included age, sex, in-hospital levels of CRP, eGFR and need for oxygen supplementation, and level of post-exertional symptoms at 1 month after discharge (fatigue and dyspnea in 6MWT and MRC dyspnea score).

## Data availability statement

The raw data supporting the conclusions of this article will be made available by the authors, without undue reservation.

## Ethics statement

The studies involving humans were approved by the Ethical committee of Kharkiv National Medical University. The studies were conducted in accordance with the local legislation and institutional requirements. The participants provided their written informed consent to participate in this study.

## Author contributions

OH: Conceptualization, Data curation, Formal analysis, Investigation, Methodology, Software, Writing – original draft, Writing – review & editing. TA: Conceptualization, Supervision, Writing – review & editing. TC: Writing – review & editing. DC: Writing – review & editing. AB: Writing – review & editing. VB: Writing – review & editing. EK: Writing – review & editing. NM: Writing – review & editing. TA: Writing – review & editing. NH: Writing – review & editing. OK: Writing – review & editing. VS: Writing – review & editing.
