# Supplementary material for: A prognostic model and pre-discharge predictors of post-COVID-19 syndrome after hospitalization for SARS-CoV-2 infection
Source: Front Public Health. 2023 Nov 29;11:1276211. doi: 10.3389/fpubh.2023.1276211 (PMC10716462; doi:10.3389/fpubh.2023.1276211)
Supplement: Supplementary file 1 [file Data_Sheet_1.pdf]

Supplementary Material

A Prognostic Model and Pre-Discharge Predictors of Post-COVID-19 Syndrome After Hospitalization for SARS-CoV-2 Infection

Oleksii Honchar*, Tetiana Ashcheulova, Tetyana Chumachenko, Dmytro Chumachenko, Alla Bobeiko, Viktor Blazhko, Eduard Khodosh, Nataliia Matiash, Tetiana Ambrosova, Nina Herasymchuk, Oksana Kochubiei, Viktoriia Smyrnova

*** Correspondence:**
Oleksii Honchar at ov.honchar@knmu.edu.ua

2 Material and Methods

Study exclusion criteria:

- stage D chronic heart failure;
- acute heart failure;
- history of myocardial infarction;
- permanent atrial fibrillation;
- stroke within 6 months;
- severe uncontrolled hypertension;
- significant valvular heart disease;
- active cancer or systemic autoimmune pathology;
- inability to provide an informed consent;
- persisting O2 supplementation dependence by the time of discharge.

3 Results

The source file of the final machine learning classification model is available in open access at https://doi.org/10.5281/zenodo.8395451 and requires an input dataset with the following variables:

- Age = age (years);
- Sex = sex (2=Female, 1=Male);
- Tx-O2 = oxygen supplementation during treatment (2=Yes, 1=No);
- CRP = peak C-reactive protein during hospitalization, mg/L;
- eGFR = estimated glomerular filtration rate by CKD-EPI equation, mL/min/1,73m^2;
- Dyspnea after 6MWT, Visit 2 = Dyspnea at the end of the 6-minute walk test 1 month post-discharge as assessed using modified Borg scale (0-10 pts);
- Fatigue after 6MWT, Visit 2 = Fatigue at the end of the 6-minute walk test 1 month post-discharge as assessed using modified Borg scale (0-10 pts) (1);
- MRC Dyspnea score, Visit 2 = MRC Dyspnea score at 1 month post-discharge.

The output codes for predicted status at 3 months post-discharge include “1” for absence and “2” for presence of the post-COVID-19 syndrome.

Supplementary Tables and Figures

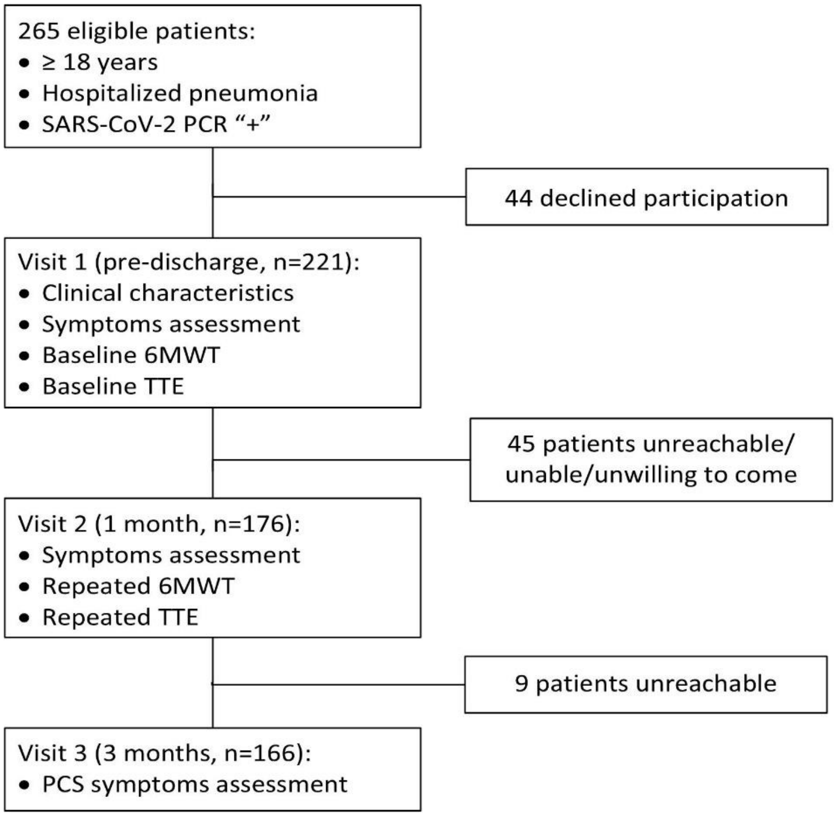

Supplementary figure 1. Study flowchart.

Supplementary table 1. Baseline marginal analysis of potential pre-discharge predictors of post-COVID-19 syndrome development in logistic regression analysis.

Effect	PCS3 - Marginal table					
	Level of effect	Somers' D	Estimate	P-value	df	Sample
Age		0,211214	0,0353051392	0,00746037481	1	Full
Pulmonary affection by CT*		0,123543	-0,016850092	0,148151789	1	Full
Minimal Sp02 during disease		0,184332	-0,0497851417	0,0763553396	1	Full
Charlson’s comorbidities score		0,234255	0,991439571	0,0083475492	1	Full
Height		0,232631	-0,042856633	0,0286682307	1	Full
Weight		0,112903	0,0108475113	0,286119087	1	Full
Body mass index		0,259953	0,0964129062	0,0144630715	1	Full
Peak D-dimer		0,08502	-0,0000650234058	0,87979646	1	Full
Peak Interleukin-6		0,096525	0,0253477288	0,0514260711	1	Full
Peak C-reactive protein		0,327303	0,00967831673	0,0594446789	1	Full
Peak Procalcitonin		-0,05303	0,189372128	0,613286403	1	Full
Hemoglobin		0,119444	-0,0128866177	0,339403843	1	Full
White blood cells		-0,047407	-0,0475919645	0,315942936	1	Full
Erythrocyte sedimentation rate		0,272464	0,0343917341	0,0467612988	1	Full
Peak Creatinine		0,149451	0,0126174065	0,285036328	1	Full
Peak Alanine transferase		0,005556	0,00734430261	0,242493654	1	Full
Peak Ferritin		0,127273	0,00095890745	0,240346255	1	Full
Lowest eGFR		0,415385	-0,0493303205	0,000652799238	1	Full
6-minute walk distance, m		0,581818	-0,0159770314	0,000045605615	1	Full
6-minute walk distance, %		0,021212	-0,000396197998	0,985685869	1	Full
Maximal HR in 6MWT, %		-0,066667	-0,00697193139	0,731922199	1	Full
HR increment in 6MWT, bpm		0,330948	-0,0396608003	0,0438441792	1	Full
Minimal SpO2 in 6MWT, %		0,162121	-0,060628245	0,293753476	1	Full
SpO2 dip in 6MWT, %		0,075758	0,0880619085	0,315974719	1	Full
Systolic blood pressure		0,309896	0,0326923437	0,0189235919	1	Full
Diastolic blood pressure		0,0625	-0,00523709716	0,771595311	1	Full
Baseline HR		0,097187	0,0139665737	0,385349632	1	Full
Baseline SpO2		0,083333	-0,00495635455	0,886494434	1	Full
Left atrial volume index		-0,004926	0,0013166046	0,961187614	1	Full
Interventricular septum		0,005376	-0,246695783	0,823033092	1	Full
LV posterior wall		0,019201	-0,482974496	0,716434089	1	Full
LV relative walls thickness		0,056068	2,59150132	0,296601199	1	Full
MAPSE		0,04827	-0,0397910466	0,960952948	1	Full
LV global longitudinal strain		0,189435	0,119667361	0,138141386	1	Full

Supplementary table 1 (continued)

LV myocardial mass		0,112903	-0,00645661088	0,150271581	1	Full
LV mass index (body surface area)		0,149102	-0,0189221195	0,0596481033	1	Full
LV mass index (height^{2,7})		-0,052303	-0,00813079221	0,672445405	1	Full
LV ejection fraction		0,013825	0,00333556713	0,898907112	1	Full
LV midwall shortening		0,003072	-0,0569341311	0,50130915	1	Full
LV end-diastolic volume index		0,263076	-0,0454718836	0,0230279757	1	Full
LV end-systolic volume index		0,164715	-0,0615198954	0,0957449819	1	Full
LV stroke volume index		0,177205	-0,0539089423	0,0508722456	1	Full
RV size		0,159439	0,755128466	0,240791991	1	Full
TAPSE		0,141383	0,24118357	0,60134542	1	Full
RV free wall strain		0,298246	0,0876764956	0,0105980102	1	Full
Right atrial size index		0,080952	-0,691287493	0,394412558	1	Full
Right atrial area index		-0,070175	0,010758537	0,907476663	1	Full
RV S'		0,2025	-0,0911770906	0,198115023	1	Full
RV E'		0,03875	-0,0292944135	0,72803251	1	Full
RV E/E'		0,02875	0,145799602	0,302675683	1	Full
LV S'		0,067588	-0,0279038243	0,785549824	1	Full
LV E'		0,206605	-0,140582377	0,0725060784	1	Full
LV E/E'		0,049155	0,0305173433	0,76113706	1	Full
LV E/E'S'		0,044547	0,256909188	0,696958439	1	Full
LV E/GLS'		0,220401	-0,340245913	0,0790969599	1	Full
COPD assessment test score		0,491228	0,134573548	0,000012542665	1	Full
Clinical COPD questionnaire score		0,233333	0,0321261771	0,0399606003	1	Full
Sex	1	0,215822	-0,438774952	0,0167091672	1	Full
Treatment - Methylprednisolone	1	0	-3,66248385E-17	1	1	Full
Treatment - Dexamethasone	1	0,097542	0,611332469	0,111432278	1	Full
Treatment - Remdesivir	1	0,071429	0,143841036	0,436111458	1	Full
Oxygen supplementation	1	0,235714	-0,480465756	0,00922390438	1	Full
CPAP	1	0,06134	0,549306144	0,133131027	1	Full
Smoking	1	0,061444	0,231311761	0,330668673	1	Full
Diabetes mellitus	1	0,065284	-0,467154618	0,2298761	1	Full
Hypertension	1	0,069892	-0,150552546	0,422057179	1	Full
Obesity	1	0,171741	-0,399253848	0,0494184938	1	Full
LV concentric geometry	1	0,041475	-0,0859251285	0,635201977	1	Full
LV hypertrophy	1	0,013825	0,0588915178	0,819425371	1	Full
LV diastolic dysfunction, grade 1	1	0,051769	0,434883775	0,25705997	2	Full
LV diastolic dysfunction, grade 2	2	0,051769	0,358897868	0,378136511	2	Full
Isolated LV E’ decrease	1	0,071429	-0,143841036	0,424008545	1	Full

	1	0,071429	-0,143841036	0,424008545	1	Full
MRC dyspnea, class 1	1	0,308653	-4,28565641	0	4	Full
MRC dyspnea, class 2	2	0,308653	-3,59250923	0	4	Full
MRC dyspnea, class 3	3	0,308653	-3,03289344	2,22044605E-16	4	Full
MRC dyspnea, class 4	4	0,308653	13,9439525		4	Full

Note. * Assessment was performed using the methodology for the simplified RALE score as proposed by Wong et al. (2), mean value of the reported % range was taken for analysis. CT – computed tomography, SpO2 – capillary blood oxygen saturation, eGFR – estimated glomerular filtration rate by CKD-EPI equation, HR – heart rate, 6MWT – 6-minute walk test, LV – left ventricle MAPSE – mitral annular plane systolic excursion, RV – right ventricle, TAPSE – tricuspid annular plane systolic excursion, COPD – chronic obstructive pulmonary disease, CPAP – constant positive airways pressure.

Supplementary table 2. Parameters of the logistic regression model to predict post-COVID-19 syndrome development.

Effect	PCS (3 months) - Parameter estimates<br>Distribution: BINOMIAL, Link function: LOGIT<br>Modeled probability that PCS3 = Yes						
	Level of Effect	Estimate	Standard Error	Wald Stat.	Lower CL 95,0%	Upper CL 95,0%	p
Intercept		-62,3586	30,51259	4,176715	-122,162	-2,5550	0,040983
BMI, kg/m^2		1,4753	0,65850	5,019646	0,185	2,7660	0,025061
ESR, mm		0,5098	0,22378	5,190114	0,071	0,9484	0,022716
eGFR, ml/min/1,73m^2		-0,4661	0,19059	5,980856	-0,840	-0,0926	0,014462
Relative LV walls thickness		68,1722	31,64079	4,642159	6,157	130,1870	0,031196
MAPSE, mm		20,9886	10,65922	3,877177	0,097	41,8803	0,048947
LV mass index, g/m^{2,7}		-0,4736	0,23569	4,038083	-0,936	-0,0117	0,044484
Scale		1,0000	0,00000		1,000	1,0000	

Note. CL – confidence limit, BMI – body mass index, ESR – erythrocyte sedimentation rate, eGFR – estimated glomerular filtration rate by CKD-EPI equation, LV – left ventricle, MAPSE – mitral annular plane systolic excursion.

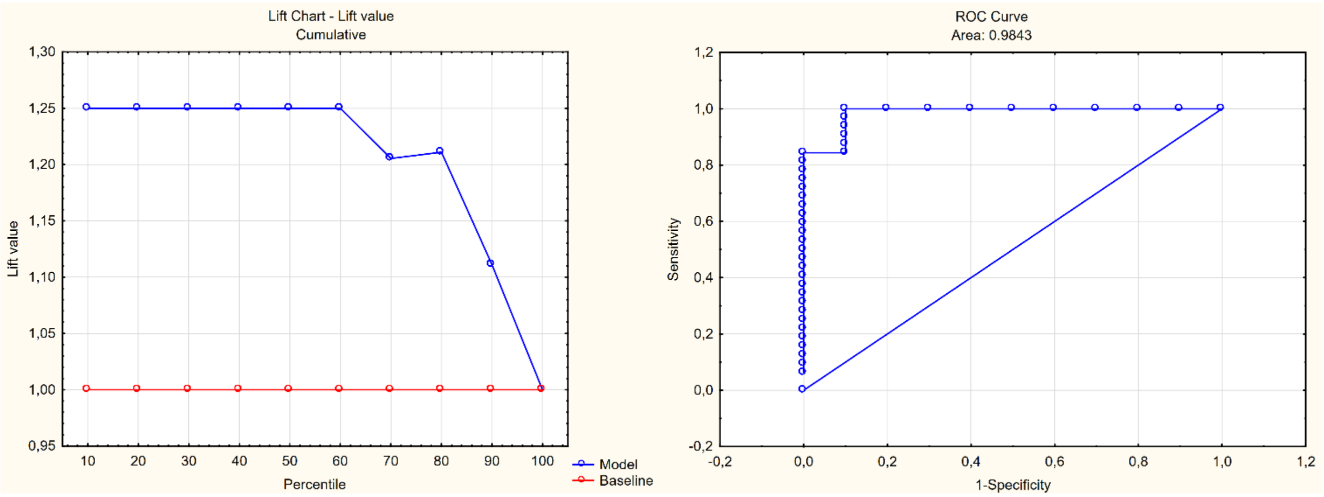

Supplementary figure 2. Lift chart (left panel) and ROC curve (right panel) of the logistic regression model to predict post-COVID-19 syndrome development.

Somers' D statistic = 0,969, Kolmogorov-Smirnov statistic = 0,900, AUROC = 0,984.

Supplementary table 3. Baseline marginal analysis of potential post-discharge (Visit 2) predictors of post-COVID-19 syndrome development in logistic regression analysis.

Effect	PCS3 - Marginal table					
	Level of effect	Somers' D	Estimate	P-value	df	Sample
6-minute walk distance, m		0,290807	-0,00856627274	0,0160604441	1	Full
6-minute walk distance, %		0,335835	0,0694802438	0,00442199449	1	Full
6-minute walk distance increment, m		0,372141	0,017294295	0,00617917837	1	Full
6-minute walk distance increment, %		0,505198	0,148011402	0,0004937592	1	Full
Maximal HR in 6MWT, %		0,4	0,0716125672	0,0131059168	1	Full
HR increment in 6MWT, bpm		0,136538	-0,0101148645	0,604003107	1	Full
Minimal SpO2 in 6MWT, %		-0,040385	0,0201968625	0,834866161	1	Full
SpO2 dip in 6MWT, %		-0,011538	-0,0656493173	0,517065631	1	Full
Dyspnea at the end of 6MWT		0,659933	0,8544486	0,000228495903	1	Full
Fatigue at the end of 6MWT		0,673401	1,1328621	0,000539787192	1	Full
Systolic blood pressure		0,401361	0,0654977184	0,00239553847	1	Full
Diastolic blood pressure		0,17517	0,0358172198	0,0884276739	1	Full
Baseline HR		0,191638	0,0195084999	0,238235091	1	Full
Baseline SpO2		0,142857	-0,438550977	0,156236321	1	Full
Baseline dyspnea		0,290941	0,617622246	0,0303398776	1	Full
Baseline fatigue		0,341463	0,501416122	0,017838572	1	Full
Left atrial volume index		-0,004255	-0,00690750918	0,835549829	1	Full
Interventricular septum		0,080556	0,0968158767	0,950454979	1	Full
LV posterior wall		0,013889	-0,0469040539	0,976146385	1	Full
LV relative walls thickness		0,002778	1,43605476	0,603842595	1	Full
MAPSE		0,066667	-0,573035066	0,512128286	1	Full
LV global longitudinal strain		0,086626	0,0389244775	0,712599279	1	Full
LV myocardial mass		0,030556	-0,00358622608	0,551897445	1	Full
LV mass index (body surface area)		0,044444	-0,00507371748	0,765910869	1	Full
LV mass index (height^{2,7})		0,227778	0,0650287254	0,059255321	1	Full
LV ejection fraction		0,080556	0,00816709837	0,789239379	1	Full
LV midwall shortening		-0,033333	-0,0389252484	0,715225957	1	Full
LV end-diastolic volume index		0,072222	-0,0182244207	0,425452456	1	Full
LV end-systolic volume index		0,094444	-0,0166119313	0,64031369	1	Full
LV stroke volume index		0,066667	-0,0335556011	0,395437079	1	Full
RV size		0,025641	0,0765079651	0,892764535	1	Full
TAPSE		0,169444	0,950448523	0,127587231	1	Full
RV free wall strain		0,338889	0,107796933	0,00710155822	1	Full
Right atrial size index		0,081159	-0,595819856	0,551124639	1	Full
Right atrial area index		0,138889	-0,185594668	0,257338744	1	Full

RV S'		0,222789	-0,121534533	0,126556426	1	Full
RV E'		0,083333	-0,102595985	0,332817646	1	Full
RV E/E'		0,081882	-0,124194717	0,529978938	1	Full
LV S'		0,118056	-0,194115623	0,127385032	1	Full
LV E'		0,2125	-0,157927962	0,0292484228	1	Full
LV E/E'		0,264881	0,293134011	0,0227141348	1	Full
LV E/E'S'		0,254464	1,74390944	0,0310678759	1	Full
LV E/GLS'		-0,06079	0,027563123	0,902093073	1	Full
COPD assessment test score		0,513141	0,146810375	0,000244159434	1	Full
Clinical COPD questionnaire score		0,416771	0,0991843974	0,000882180343	1	Full
MRC dyspnea, class 1	1	0,329412	-0,97068495	0,00357528775	2	Full
MRC dyspnea, class 2	2	0,329412	-0,0639636693	0,872833093	2	Full

Note. HR – heart rate, 6MWT – 6-minute walk test, SpO2 – capillary blood oxygen saturation, LV – left ventricle MAPSE – mitral annular plane systolic excursion, RV – right ventricle, TAPSE – tricuspid annular plane systolic excursion, COPD – chronic obstructive pulmonary disease.

Supplementary table 4. Connections and weight values of the final machine learning model predicting post-COVID-19 syndrome at 3 months post-discharge.

Weight ID	Connections	Weight values
1	Age --> hidden neuron 1	1,41383
2	CRP --> hidden neuron 1	0,49931
3	eGFR --> hidden neuron 1	-1,36148
4	Dyspnea after 6MWT, Visit 2 --> hidden neuron 1	1,25080
5	Fatigue after 6MWT, Visit 2 --> hidden neuron 1	1,50884
6	Sex(1) --> hidden neuron 1	0,13656
7	Sex(2) --> hidden neuron 1	0,31986
8	Tx-O2(1) --> hidden neuron 1	-0,64534
9	Tx-O2(2) --> hidden neuron 1	1,12150
10	MRC Dyspnea score, Visit 2(1) --> hidden neuron 1	-0,56965
11	MRC Dyspnea score, Visit 2(1,5000000000) --> hidden neuron 1	-0,63012
12	MRC Dyspnea score, Visit 2(2) --> hidden neuron 1	0,11891
13	MRC Dyspnea score, Visit 2(3) --> hidden neuron 1	0,39226
14	Age --> hidden neuron 2	-0,73558
15	CRP --> hidden neuron 2	0,29652
16	eGFR --> hidden neuron 2	0,00513
17	Dyspnea after 6MWT, Visit 2 --> hidden neuron 2	0,21280
18	Fatigue after 6MWT, Visit 2 --> hidden neuron 2	0,10636
19	Sex(1) --> hidden neuron 2	-0,07579
20	Sex(2) --> hidden neuron 2	0,27979
21	Tx-O2(1) --> hidden neuron 2	-0,58048
22	Tx-O2(2) --> hidden neuron 2	0,75607
23	MRC Dyspnea score, Visit 2(1) --> hidden neuron 2	-0,19822
24	MRC Dyspnea score, Visit 2(1,5000000000) --> hidden neuron 2	-0,19646
25	MRC Dyspnea score, Visit 2(2) --> hidden neuron 2	0,01663
26	MRC Dyspnea score, Visit 2(3) --> hidden neuron 2	0,26422
27	Age --> hidden neuron 3	1,29153
28	CRP --> hidden neuron 3	-0,14712
29	eGFR --> hidden neuron 3	-0,55013
30	Dyspnea after 6MWT, Visit 2 --> hidden neuron 3	0,39581
31	Fatigue after 6MWT, Visit 2 --> hidden neuron 3	0,16721
32	Sex(1) --> hidden neuron 3	0,36412
33	Sex(2) --> hidden neuron 3	-0,10535
34	Tx-O2(1) --> hidden neuron 3	0,04303
35	Tx-O2(2) --> hidden neuron 3	0,21551
36	MRC Dyspnea score, Visit 2(1) --> hidden neuron 3	-0,08181

37	MRC Dyspnea score, Visit 2(1,5000000000) --> hidden neuron 3	-0,14773
38	MRC Dyspnea score, Visit 2(2) --> hidden neuron 3	-0,08557
39	MRC Dyspnea score, Visit 2(3) --> hidden neuron 3	0,32082
40	Age --> hidden neuron 4	-2,31260
41	CRP --> hidden neuron 4	-0,27158
42	eGFR --> hidden neuron 4	3,97941
43	Dyspnea after 6MWT, Visit 2 --> hidden neuron 4	-0,40737
44	Fatigue after 6MWT, Visit 2 --> hidden neuron 4	-1,61592
45	Sex(1) --> hidden neuron 4	0,11001
46	Sex(2) --> hidden neuron 4	-0,24714
47	Tx-O2(1) --> hidden neuron 4	0,01062
48	Tx-O2(2) --> hidden neuron 4	-0,21526
49	MRC Dyspnea score, Visit 2(1) --> hidden neuron 4	0,16930
50	MRC Dyspnea score, Visit 2(1,5000000000) --> hidden neuron 4	0,22489
51	MRC Dyspnea score, Visit 2(2) --> hidden neuron 4	0,30722
52	MRC Dyspnea score, Visit 2(3) --> hidden neuron 4	0,00592
53	Age --> hidden neuron 5	-1,40131
54	CRP --> hidden neuron 5	0,42530
55	eGFR --> hidden neuron 5	-0,91092
56	Dyspnea after 6MWT, Visit 2 --> hidden neuron 5	1,90255
57	Fatigue after 6MWT, Visit 2 --> hidden neuron 5	1,34225
58	Sex(1) --> hidden neuron 5	0,44057
59	Sex(2) --> hidden neuron 5	-0,51699
60	Tx-O2(1) --> hidden neuron 5	-0,49116
61	Tx-O2(2) --> hidden neuron 5	0,46070
62	MRC Dyspnea score, Visit 2(1) --> hidden neuron 5	0,00693
63	MRC Dyspnea score, Visit 2(1,5000000000) --> hidden neuron 5	-0,04329
64	MRC Dyspnea score, Visit 2(2) --> hidden neuron 5	0,30638
65	MRC Dyspnea score, Visit 2(3) --> hidden neuron 5	-0,48393
66	Age --> hidden neuron 6	0,80230
67	CRP --> hidden neuron 6	-0,10221
68	eGFR --> hidden neuron 6	0,04898
69	Dyspnea after 6MWT, Visit 2 --> hidden neuron 6	-1,64203
70	Fatigue after 6MWT, Visit 2 --> hidden neuron 6	-0,98386
71	Sex(1) --> hidden neuron 6	0,03001
72	Sex(2) --> hidden neuron 6	0,12318
73	Tx-O2(1) --> hidden neuron 6	0,60731
74	Tx-O2(2) --> hidden neuron 6	-0,45472
75	MRC Dyspnea score, Visit 2(1) --> hidden neuron 6	0,57034
76	MRC Dyspnea score, Visit 2(1,5000000000) --> hidden neuron 6	0,53061
77	MRC Dyspnea score, Visit 2(2) --> hidden neuron 6	-0,67563

Supplementary table 4 (continued)

78	MRC Dyspnea score, Visit 2(3) --> hidden neuron 6	0,36668
79	Age --> hidden neuron 7	0,74909
80	CRP --> hidden neuron 7	0,44444
81	eGFR --> hidden neuron 7	-0,52140
82	Dyspnea after 6MWT, Visit 2 --> hidden neuron 7	0,69082
83	Fatigue after 6MWT, Visit 2 --> hidden neuron 7	0,67769
84	Sex(1) --> hidden neuron 7	-0,28853
85	Sex(2) --> hidden neuron 7	0,23535
86	Tx-O2(1) --> hidden neuron 7	-0,78691
87	Tx-O2(2) --> hidden neuron 7	0,73578
88	MRC Dyspnea score, Visit 2(1) --> hidden neuron 7	-0,95047
89	MRC Dyspnea score, Visit 2(1,5000000000) --> hidden neuron 7	-0,94325
90	MRC Dyspnea score, Visit 2(2) --> hidden neuron 7	-0,15917
91	MRC Dyspnea score, Visit 2(3) --> hidden neuron 7	0,86226
92	input bias --> hidden neuron 1	0,49218
93	input bias --> hidden neuron 2	0,18388
94	input bias --> hidden neuron 3	0,27142
95	input bias --> hidden neuron 4	-0,23790
96	input bias --> hidden neuron 5	-0,09347
97	input bias --> hidden neuron 6	0,09126
98	input bias --> hidden neuron 7	0,03987
99	hidden neuron 1 --> PCS (3 months)(1)	-0,78338
100	hidden neuron 2 --> PCS (3 months)(1)	0,08600
101	hidden neuron 3 --> PCS (3 months)(1)	0,69153
102	hidden neuron 4 --> PCS (3 months)(1)	0,45268
103	hidden neuron 5 --> PCS (3 months)(1)	-1,10845
104	hidden neuron 6 --> PCS (3 months)(1)	-0,45718
105	hidden neuron 7 --> PCS (3 months)(1)	-0,81890
106	hidden neuron 1 --> PCS (3 months)(2)	-0,01074
107	hidden neuron 2 --> PCS (3 months)(2)	-0,19221
108	hidden neuron 3 --> PCS (3 months)(2)	-0,19820
109	hidden neuron 4 --> PCS (3 months)(2)	-0,61698
110	hidden neuron 5 --> PCS (3 months)(2)	0,27186
111	hidden neuron 6 --> PCS (3 months)(2)	0,00964
112	hidden neuron 7 --> PCS (3 months)(2)	0,10255
113	hidden bias --> PCS (3 months)(1)	0,69739
114	hidden bias --> PCS (3 months)(2)	0,51890

Note. Training algorithm = BFGS 33; Error function = SOS; Hidden activation = Exponential; Output activation = Exponential.

References

1. Borg GA. Psychophysical bases of perceived exertion. Med Sci Sports Exerc. 1982;14(5):377-81.

2. Wong HYF, Lam HYS, Fong AH, Leung ST, Chin TW, Lo CSY, et al. Frequency and Distribution of Chest Radiographic Findings in Patients Positive for COVID-19. Radiology. 2020;296(2):E72-E8.
